# Maternal omega-3 polyunsaturated fatty acid supplementation on offspring hip joint conformation

**DOI:** 10.1371/journal.pone.0202157

**Published:** 2018-08-09

**Authors:** A. M. Oberbauer, R. Daniels, K. Levy, T. R. Famula, P. Mundell, R. Kelley

**Affiliations:** 1 Department of Animal Science, University of California, Davis, CA, United States of America; 2 Canine Companions for Independence, Santa Rosa, CA, United States of America; 3 The Iams Company, P&G Pet Care, Mason, Ohio, United States of America; University of Illinois, UNITED STATES

## Abstract

Unsaturated omega-3 fatty acids, especially docosahexaenoic acid (DHA), when fed to dogs improves cognitive and neurological development. Supplementation with omega-3 fatty acids such as DHA and eicosapentaenoic acid (EPA) has also been associated with lipid peroxidation, which in turn has been implicated in reduced body weight and altered bone formation. To assess the impact of omega-3 fatty acid supplementation on skeletal growth, diets containing three levels of DHA and EPA (0.01 and 0.01%, 0.14 and 0.12%, and 0.21 and 0.18%, respectively) were fed to bitches during gestation and lactation with puppies also supplemented through weaning. Thus, the subjects studied were the puppies supplemented with DHA and EPA through gestation and early postnatal life. The hip joint conformation of the puppies (n = 676) was recorded at adulthood using two radiographic, non-invasive evaluations. In this population, females had higher hip distraction indices (DI) than males. Males from the lower two levels of DHA and EPA supplementation had significantly smaller hip DI than all females and males from the highest DHA and EPA supplementation. In contrast, there were no diet effects on anatomical indicators of hip joint conformation and no visible arthritic changes. These data suggest that dietary supplementation of DHA and EPA during gestation and the perinatal period to weaning does not adversely influence hip joint formation of dogs.

## Introduction

Fatty acids are recognized as important dietary components in human health with omega-3 fatty acids suggested as playing a significant role in cardiovascular health [1, 2]. Furthermore, omega-3 fatty acids are necessary for normal brain development and maintenance of cognitive function [3, 4] as well as having a role in muscle accretion among the aged [5] and mitigation of inflammatory pain associated with rheumatoid arthritis and other autoimmune conditions [6, 7]. Recent meta-analyses and large well-controlled clinical trials have questioned omega-3 fatty acids as a therapeutic for coronary vascular disease [8] and cognitive function in the elderly [9] but have validated the role as an analgesic for joint pain [10].

Human research has identified the omega-3 polyunsaturated fatty acid docosahexaenoic acid (DHA), C22:6n-3, as an essential nutrient playing a key role during growth and development [11] leading to recommendations of dietary supplementation of omega-3 fatty acids. Such recommendations have led to multiple studies in growing and aged dog populations. Similar to results obtained in human studies, supplementing adult dogs with omega-3 fatty acids elevated serum alpha-linolenic acid (ALA), DHA, and eicosapentaenoic acid (EPA) and ameliorated pain related to osteoarthritis [12, 13], perhaps through a reduction in arachidonic acid [14]. Feeding puppies omega-3 -enriched foods improved cognitive development as assessed by memory and maze navigation [15] and supplementing pregnant and lactating bitches with omega-3 fatty acids is associated with increased retinal function, cognitive ability, and trainability of the puppies [16–18] with DHA being more effective than ALA [18]. Thus, feeding omega-3 fatty acids to dogs of all ages has been suggested to be generally valuable [19–22].

In a long term, 9 month study, DHA demonstrated no overt signs of toxicity when fed to 6-7month old beagles dogs [23] and another study reported that dogs were able to tolerate high dietary DHA levels without intestinal upset [14]. Nevertheless, investigators have urged caution in that potential risks of DHA supplementation have not been fully explored [23, 24] and adverse responses to dogs fed omega-3 fatty acids, catalogued in Lenox and Bauer [24], include gastric upset, lipid peroxidation, impaired immune function, and prolonged wound healing.

Increased lipid peroxidation has been associated with reduced body weight and bone formation in adolescent dogs [25] and reduced body fat accrual [15, 25] and cartilage synthesis protein II [15]. Importantly, when fed elevated levels of oxidized dietary fatty acids, 8-week old pups exhibited reduced weight gain and body fat, lowered circulating vitamin E, and impaired bone formation with reduced trabecular bone volume [25]. Work in poultry and rats have demonstrated a positive correlation with circulating vitamin E and bone mineral density, trabecular volume, and linear elongation [26, 27] suggesting that any alteration in vitamin E, such as through lipid peroxidation, could impact overall bone development.

In humans, maternal intake of dietary omega-3 fatty acids during pregnancy was associated with reduced bone growth and shorter stature of the children at later ages [28]. In contrast, direct dietary supplementation of omega-3 fatty acids correspond to reduced bone turnover with inhibition of osteoclast function and promotion of bone deposition through enhanced osteoblast formation (reviewed in [29]). Elevated omega-3 fatty acids during the active growth phase in mice increased the proliferation and differentiation of the growth plate resulting in increased linear bone elongation [30]. These differing reports suggest that omega-3 fatty acids may exert species-specific influences on bone.

Given the favorable impact of unsaturated omega-3 fatty acids on cognitive and neurological development in the dog, the evaluation of DHA and EPA supplementation on other physiological systems is warranted, especially effects on bone and joint formation. Disrupted bone growth, either accelerated or inhibited, may be manifested in the dog as joint dysplasia. The present study examined the effects of supplementing pregnant and lactating bitches with dietary DHA and EPA on the development of hip joint formation using standard, non-invasive modes of evaluation of joint conformation for dogs.

## Materials and methods

### Study population

All dogs were part of Canine Companions for Independence (CCI), a non-profit organization in Santa Rosa, California that breeds, trains, and places assistance dogs to aid people with disabilities. The study was approved by The Iams Company IACUC and the CCI Research Committee and was compliant with the CCI’s scientific oversight committee for animal care. Golden retrievers (n = 19), Labrador retrievers (n = 32), and Labrador-golden retriever cross (n = 52) dams were fed a nutritionally complete diet supplemented with one of three levels of DHA and EPA: a low diet containing 0.01% of both DHA and EPA; a moderate diet containing 0.14% DHA and 0.12% EPA; and a high diet containing 0.21% DHA and 0.18% EPA. The ratio of DHA and EPA was kept relatively constant in each of the three diets in accordance with the National Research Council (NRC) recommendation for dogs [31]. The study ran over two years. For each diet, ingredient lots were held constant. Diets were produced four times during the course of the study and the diet composition analyzed at production and at end of use for each production run: proximate analyses were performed in triplicate and amino acids, vitamins, minerals, and fatty acids analyses were performed in duplicate. The average analytical composition of the diets fed is presented in Table 1.

**Table 1:** Average analytical composition of the supplemental diets (see text for analytical details).

		Diet		
Nutrient	Units	Low ^†^	Moderate	High
**Moisture**	%	7.97	8.10	7.95
**Ash**	%	6.50	6.53	6.61
Calcium	%	1.27	1.2	1.3
Copper	ppm	20.51	19.83	20.18
Iron	ppm	330.49	328.36	357.93
Potassium	%	0.65	0.65	0.69
Magnesium	ppm	867.31	846.35	914.43
Manganese	ppm	48.28	48.7	48.79
Sodium	ppm	4405	4404	4626
Phosphorus	%	1.02	0.98	1.06
Zinc	ppm	238.13	226.25	238.91
**Protein**	%	31.32	31.73	31.39
Cystine	%	0.713	0.667	0.671
Arginine	%	2.209	2.494	2.195
Taurine	%	0.118	0.15	0.133
Hydroxyproline	%	0.623	0.951	0.744
Serine	%	1.208	1.476	1.46
Aspartic Acid	%	2.379	2.806	2.694
Glutamic Acid	%	3.955	4.596	4.453
Threonine	%	1.17	1.378	1.337
Glycine	%	1.976	2.656	2.341
Alanine	%	1.751	2.121	2.007
Proline	%	1.541	1.918	1.77
Methionine	%	0.725	0.852	0.833
Valine	%	1.3	1.313	1.272
Phenylalanine	%	1.131	1.327	1.29
Isoleucine	%	1.176	1.226	1.171
Leucine	%	2.122	2.435	2.418
Histidine	%	0.409	0.593	0.883
Lysine	%	1.766	2.06	1.968
Tyrosine	%	0.762	0.947	0.889
Tryptophan	%	0.258	0.255	0.297
**Fat**	%	21.51	20.90	21.01
Palmitic	%	4.93	4.73	4.76
Oleic	%	7.97	7.36	7.23
Linoleic	%	4.23	3.98	3.95
Alpha-linolenic	%	0.61	0.32	0.19
Eicosapentaenoic	%	0.01	0.12	0.18
Docosahexaenoic	%	0.01	0.14	0.21
**Total Unsaturated Omega-3**	%	0.65	0.67	0.72
**Total Unsaturated Omega-6**	%	4.56	4.31	4.29
**Omega-6:3 Ratio**		7.02:1	6.43:1	5.96:1
**Vitamins**				
Biotin	mg/kg	0.75	0.73	0.58
Niacin	mg/kg	71.9	74.1	69.1
Pantothenic Acid	mg/kg	44.2	50.8	44.9
Thiamine	mg/kg	26.2	24.4	26.0
Vitamin B12	mg/kg	0.242	0.222	0.244
Riboflavin	mg/kg	22.9	17.0	17.6
Vitamin B6	mg/kg	12.9	11.8	12.5
Vitamin A	IU/kg	22006	20964	21537
Vitamin C	mg/kg	153	167	120
Vitamin D	IU/kg	1720	1620	2330
Vitamin E	IU/kg	365	370	440

† Low, moderate, and high refers to the docosahexaenoic acid (DHA) and eicosapentaenoic acid (EPA) levels in the diet

Total dietary fat was held constant across the diets, and in an effort to maintain a comparable omega-6 to omega-3 ratio, alpha linolenic acid (ALA) was added to balance the DHA and EPA; when DHA and EPA were low, ALA was proportionately high. Dams were fed the dietary treatments from breeding until the pups were weaned. At 30 days of age, puppies were introduced to their maternal diets as solid food supplementing their nursing. That is, for example, puppies born to dams supplemented with the low diet were fed the low diet as they transitioned to solid food. At weaning, the pups born to all dams were weaned to commercial diets containing a minimum of 0.05% DHA; ~83% of pups were fed Eukanuba large breed puppy post-weaning and the other 17% transitioned to other Eukanuba diets such as Lamb & Rice Puppy or Low Residue Puppy. Pups were raised by volunteer puppy raisers who were provided with identical training manuals and guidelines that covered vaccination schedule, health management, and exercise regimens. Feeding directions indicated feeding of three cups (~0.68 kg) feed per day adjusted appropriately to maintain a body condition score of 4 to 5, defined as ribs easily palpable with minimal or no excess fat covering, an obvious waist when viewed from above and evident abdominal tuck up when viewed from above. Pups were returned to the CCI facility at 18–20 months of age, at which time their hip and elbow joints were evaluated for evidence of dysplasia. Although individual growth curves for each puppy were not assessed, the median weights for male and female dogs upon return to the CCI facility at 18–20 months of age were 30.4 and 25.9 kgs, respectively.

There are two radiographic-based phenotypic methods available in the United States for evaluating and scoring of hip dysplasia: the OFA (Orthopedic Foundation for Animals) and PennHip phenotypic assessments [32, 33]. The former uses nine anatomical indicators of joint conformation and visible arthritic changes observable in ventral dorsal hip-extended radiographs to subjectively score the hips (https://www.ofa.org/diseases/hip-dysplasia/hip-screening-proceduresaccessed10/19/2017) and the latter utilizes a distraction index (DI) measuring the distance from the femoral head to the center of the acetabulum divided by the radius of the femoral head when distraction force is applied to the joint [34]. Anatomical evaluation of the elbow is the widely used assessment tool for elbow dysplasia [35] and is the OFA method used to evaluate elbow conformation.

The OFA hip categories are excellent, good, fair, borderline, mild dysplasia, moderate dysplasia, and severe dysplasia with the outcomes of both hips incorporated into a single rating encompassing both hips. Excellent, good, and fair ratings are considered non-dysplastic. Hip scores were coded for analysis: excellent = 1, good = 2, fair = 3, borderline = 4, mild dysplasia = 5, moderate dysplasia = 6, and severe dysplasia = 7. Similarly, elbow ratings were coded with normal = 1, degenerative joint disease grade I = 2, degenerative joint disease grade II = 3, and degenerative joint disease grade III = 4. PennHip ratings are unit-free indices with the possibility of ranging from 0 to 1.0 and therefore did not need additional coding for analyses. The PennHip assessment determines the DI for each hip individually. Data recorded for each pup included dam, diet, parity of the dam, sire of litter, breed, number of pups born in each litter, sex, OFA hip evaluation, PennHip index, and OFA elbow evaluation.

### Statistics

The principal objective of this work was the assessment of dietary DHA and EPA supplementation on hip development. Additionally, having dogs simultaneously evaluated under the two schemas for the diagnosis of hip dysplasia allowed for their direct comparison. We note, however, that the OFA evaluations were considered “preliminary” because final OFA ratings require dogs to be 24 months or older at time of radiography and these dogs were evaluated at 18–20 months of age. Moreover, because the dogs in the various treatment groups are related to one another, we also estimated the correlations (including genetic) between the OFA and PennHIp scoring systems. The algebraic form for this multiple trait analysis, treating each hip measurement (i.e., PennHip of the left, PennHip of the right and the ordered categorical rating of OFA) as separate and potentially correlated traits, is

$$y_{ijkmn}=\mu_{n}+\;sex_{in}+\;diet_{jn}+breed_{kn}+animal_{ijkmn}+e_{ijkmn}$$

where $y_{ijkmn}$ is the observed hip measurement for the n-th trait (n = Penn left, Penn right, OFA) of the m-th (m = 1,2,…,500) dog of the i-th (i = male, female) sex in the k-th (k = GLD, LAB or LGX) breed fed the j-th (j = high, moderate, low) diet, $\mu_{n}$ is a constant common to all observations of hip measure n, ${sex}_{in}$ is the effect of the i-th sex on hip measure n, ${diet}_{jn}$ is the effect the j-th diet on hip measure n, ${breed}_{kn}$ is the effect of the k-th breed on hip measure n, ${animal}_{ijkmn}$ is the random additive genetic contribution of the m-th dog nested in the k-th breed, j-th diet and i-th sex to hip measure n and finally, $e_{ijkmn}$ is the random residual associated with hip measure n in the m-th dog of the ijk-th sex, diet and breed. It should also be noted that the two DI measures are continuously (i.e., normally) distributed, whereas the OFA scores are ordinal. As such, the model outlined above when considered for the ordinal OFA scores is relevant to the underlying continuous variate rather than the OFA scores directly. Finally, the model outlined above is one of several investigated. Extensions of this model including interactions among effects (e.g., interaction of diet and sex) were also considered for each of the hip measurements.

If we define $a_{L},\;a_{R},\;a_{O},\;e_{L},\;e_{R}$ and $e_{O}$ as vectors of animal and residual effects for each trait, Penn left Penn right and OFA, respectively, then we can assume that each of these random variables are distributed with null means and covariance structures

$$var\left[\begin{array}{c} \begin{array}{c} a_{L}\\ a_{R} \end{array}\\ a_{O} \end{array}\right]=G_{0}⊗A=\left[\begin{array}{ccc} g_{LL} & g_{LR} & g_{LO}\\ g_{LR} & g_{RR} & g_{RO}\\ g_{LO} & g_{RO} & g_{OO} \end{array}\right]⊗A$$ (1)

and

$$var\left[\begin{array}{c} \begin{array}{c} e_{L}\\ e_{R} \end{array}\\ e_{O} \end{array}\right]=R_{0}⊗I=\left[\begin{array}{ccc} r_{LL} & r_{LR} & r_{LO}\\ r_{LR} & r_{RR} & r_{RO}\\ r_{LO} & r_{RO} & r_{OO} \end{array}\right]⊗I$$ (2)

where $g_{LL},\;g_{RR}$ and $g_{OO}$ represent the additive genetic variances in DI for the left and right hips and OFA scores, respectively, and $g_{LR},\;g_{LO}$ and $g_{RO}$ represent the additive genetic covariances between the three hip traits, A is the matrix of numerator relationships for all dogs in the pedigree (595 dogs including those without hip measurements) and ⨂ denotes the direct-product. In a similar fashion, $r_{LL},\;r_{RR}$ and $r_{OO}$ represent the residual variances in DI for the left and right hips and OFA scores, respectively, and $r_{LR},\;r_{LO}$ and $r_{RO}$ represents the residual covariances between the three hip traits, I is an identity matrix of order the number of observations on all hip traits (500 dogs with recorded hip measurements).

Accordingly, the heritability of the left PennHip measurement is $h_{L}^{2}=g_{LL}/\left(g_{LL}+r_{LL}\right)$, the heritability of the right PennHip measurement is $h_{R}^{2}=g_{RR}/\left(g_{RR}+r_{RR}\right)$, and the genetic correlation of PennHip measurements of the two sides is $r_{g\cdot LR}=g_{LR}/\sqrt{g_{LL}g_{RR}}$. This approach was also used to derive the heritability of the OFA hip assessment. Similarly, the genetic correlation between the left PennHip measurement and the OFA score was evaluated with the statistic $g_{g\cdot LO}=g_{g\cdot LO}/\sqrt{g_{LL}g_{OO}}$, as well as the genetic correlation between the right PennHip measurement and the OFA score can be evaluated as $g_{g\cdot RO}=g_{g\cdot RO}/\sqrt{g_{RR}g_{OO}}$.

Estimation of the unknown genetic parameters, along with estimates of diet and breed differences in hip measurement, was conducted with the public domain package MCMCglmm [36], a component of the widely used language R [37]. Being a package built upon a Bayesian framework, our parameter estimates were taken from a sample of 3,000 generated values in one chain, where the total number of samples was 200,000 with a burn-in period of 50,000 samples where every 50-th sample was saved as values from the posterior density.

Comparisons of hip measurements across sex and diet classifications were also facilitated from this sample of the posterior density. That is, the mean difference in hip measurements between groups (e.g. males of the high diet against males of the low diet) can be contrasted using the parameter estimates for diet and sex contributions in the 3,000 generated samples. Estimates of the 95% highest posterior density region for these differences, computed with the R-language library coda [38], were then used to evaluate if any contrasts were likely to include zero, thus providing the Bayesian equivalent of a confidence interval and its associated test of significance.

## Results

**Fig 1:**
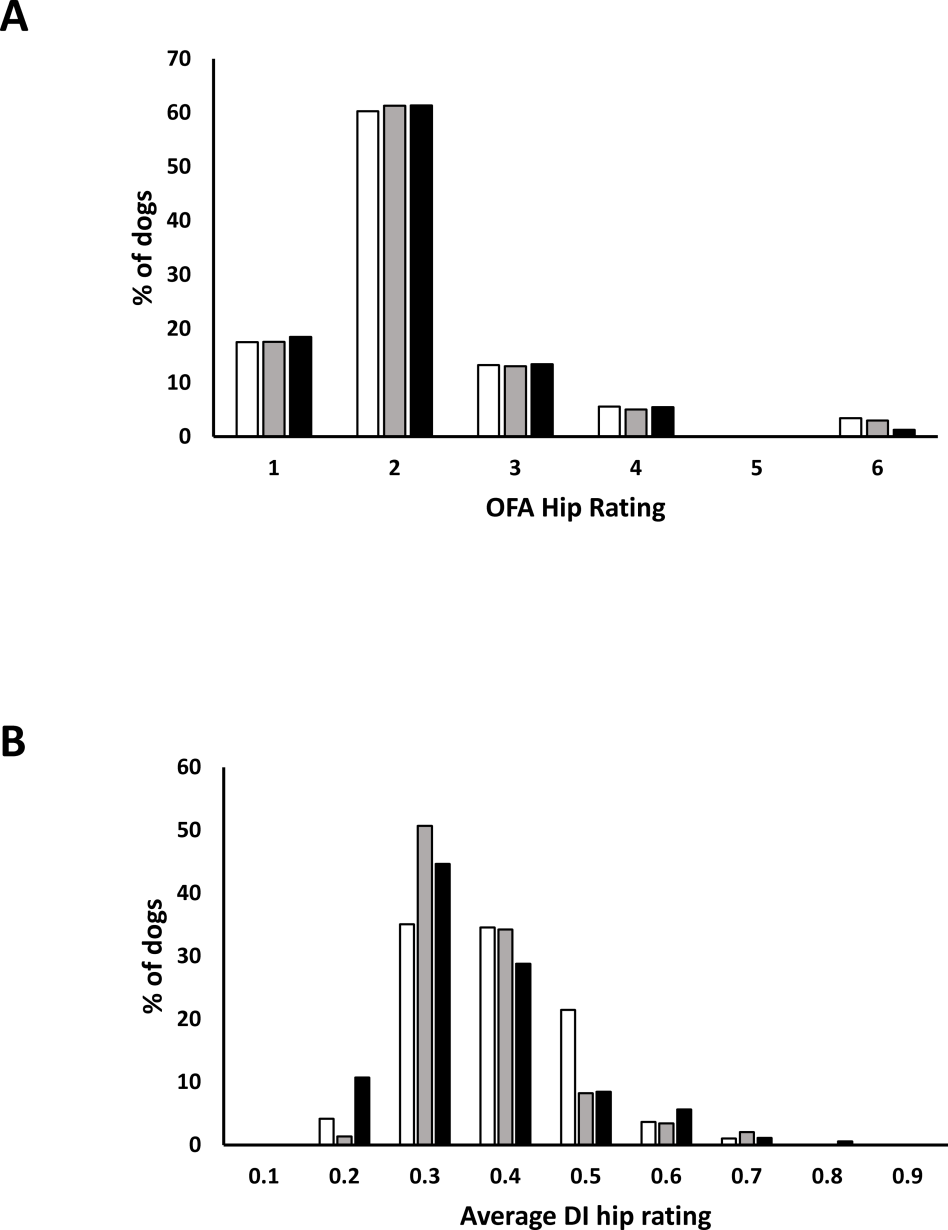
Percentage of hip evaluations as a function of diet for OFA and PennHip assessment schemes. Data expressed as a percentage of the total number of pups fed either the high (white bar), moderate (gray bar), or low (black bar) DHA and EPA diet. A) OFA hip categories with 1, 2, and 3 were considered non-dysplastic. B) PennHip Distraction Index (DI) measurements with indices presented as whole values; that is, for DI indices ranging from 0.2 to 0.299 they are presented as 0.2 in this figure.

Seventy-four omega-3 supplemented dams produced 96 litters over the course of the study with 676 pups having hip evaluation data representing 350 males, 324 females, and two without sex recorded. There were 235, 201, and 240 dogs on the high, moderate, and low diets, respectively (Table 2). Pups with elbow data numbered 652 composed of 329 males, 321 females, and two without sex recorded for 214, 201, and 237 dogs on the high, moderate, and low diets, respectively. Elbow dysplasia was relatively uncommon with only 2.5% of the pups receiving a rating consistent with degenerative joint disease; the low prevalence precluded meaningful analyses. Fig 1 depicts the hip distributions for OFA and PennHip evaluations as a function of diet. The vast majority of pups were considered non-dysplastic for hips by the OFA system having ratings of 1, 2, or 3 (92.1%) and 79.5% had an average DI of less than 0.499.

**Table 2:** Breed and sex distribution of dogs by diet with hip joint conformation evaluated^†^.

	Golden retriever		Labrador retriever		Labrador-Golden cross	
Diet	Male	Female	Male	Female	Male	Female
High	32	36	34	23	61	49
Moderate	17	19	29	29	58	48
Low	16	18	41	42	62	60

†One Labrador (low diet) and one Labrador-Golden cross (moderate diet) did not have a recorded sex in the dataset

The statistical model employed accounts for all relationships when estimating parameters including sire and dam influence along with breed, number born, and parity. Additionally, because the study ran over two years, year effect was analyzed and found not to be significant. For the OFA hip analyses, no parameters were significant, including diet (Table 3). When looking at the PennHip data, sex was a significant factor for DI values with females having greater average DI than males: 0.432 + 0.006 and 0.397 + 0.006, p < 0.0001, respectively. The average of the two PennHip DI scores for each dog showed a significant effect of diet for males only with the low and moderate diet having the lowest indices when compared to males on the high diet or female (Table 3). Both hips individually showed the same result.

**Table 3:** Estimates for hip joints of the OFA scheme and the PennHip scheme as a function of diet and sex. PennHip measures distraction index on each hip individually, thus data are presented for each hip and the average for a dog. Data are presented as the parameter estimate, standard error (SE) of the estimate, and the low and high highest posterior density (HPD) of the estimate; * indicates the estimates for a given measurement differed by diet (p < 0.05).

	OFA				Average PennHip				Left PennHip				Right PennHip				
Diet																
	Estimate	SE	Low HPD	High HPD	Estimate	SE	Low HPD	High HPD	Estimate	SE	Low HPD	High HPD	Estimate	SE	Low HPD	High HPD
**High**																
Males	2.23	0.145	1.864	2.431	0.424	0.016	0.380	0.442	0.426	0.017	0.382	0.449	0.423	0.018	0.373	0.443
Females	2.21	0.146	1.816	2.388	0.440	0.016	0.413	0.475	0.448	0.016	0.428	0.492	0.431	0.017	0.391	0.459
**Moderate**																
Males	2.10	0.144	1.604	2.167	0.387*	0.016	0.307	0.369	0.391*	0.017	0.313	0.378	0.383*	0.017	0.294	0.360
Females	2.31	0.139	2.035	2.581	0.436	0.017	0.404	0.472	0.435	0.016	0.402	0.466	0.438	0.018	0.405	0.474
**Low**																
Males	2.12	0.143	1.637	2.197	0.392*	0.017	0.314	0.381	0.396*	0.018	0.324	0.393	0.390*	0.019	0.305	0.378
Females	2.19	0.146	1.771	2.342	0.432	0.016	0.397	0.460	0.435	0.017	0.402	0.467	0.433	0.018	0.395	0.464

**Fig 2:**
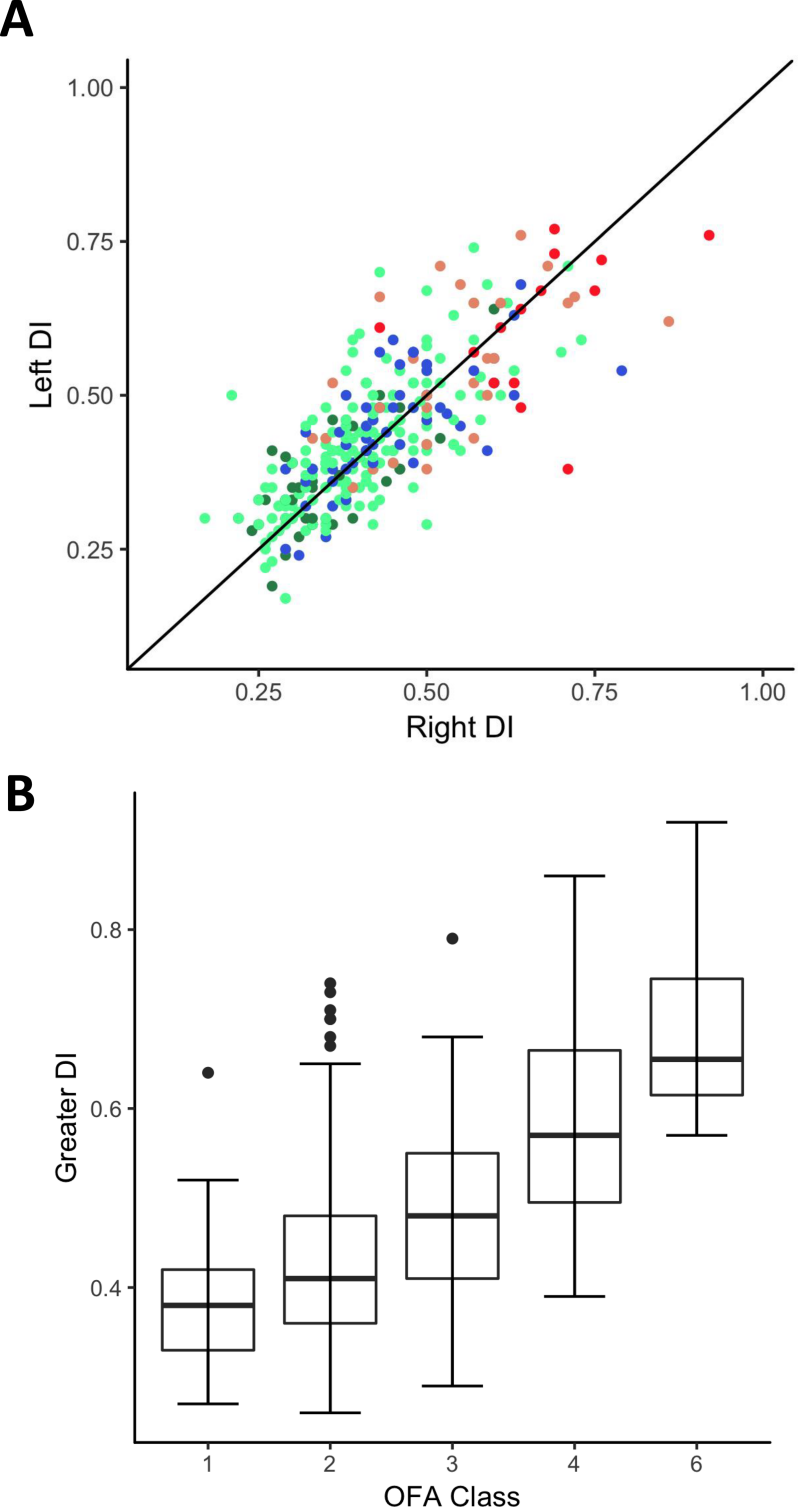
Relationship of left and right hip distraction indices (DI) with the OFA scores (A) and the relationship of the maximum DI for a given dog with its OFA rating (B). For A, OFA scores of 1 (green dots), 2 (teal dots), and 3 (blue dots) are considered non-dysplastic whereas 4 (peach dots) and 6 (red dots) are considered dysplastic.

To compare the more frequently (within the USA) used OFA hip assessment methodology to that of the PennHip methodology, the first step required confirmation that the two individual hip DI values were behaving as a single trait. The genetic correlation between the left and right PennHip DI was 0.91 (with a 95% confidence interval (CI) of 0.77 to 0.99) and the heritability of the left hip DI was 0.25 (with a 95% CI of 0.05 to 0.46), and the heritability of the right hip DI was 0.25 (with a 95% CI of 0.04 to 0.46). Thus, the two hips were behaving as essentially the same trait. Heritability of the OFA hip trait was 0.40 (with a 95% CI of 0.07 to 0.73). Each DI measure was positively correlated with the OFA category, such that the genetic correlation between the left PennHip and the OFA score was 0.49 (with a 95% CI of -0.03 to 0.93) and between the right PennHip and the OFA score was 0.54 (with a 95% CI of 0.04 to 0.95). This relationship is visualized in Fig 2 (using the observed DI and OFA measures) demonstrating that as DI increases the rating of the OFA hips rose indicating a greater degree of dysplasia.

## Discussion

The present study utilized non-invasive techniques to determine the impact of dietary supplementation of omega-3 fatty acids throughout gestation and the perinatal period on skeletal development. Hip conformation can be an indicator of disrupted bone growth in dogs [39] as growth must be coordinated among the many components that form the joint [40]. For example, trabecular bone must be aligned in the acetabulum and femoral head and be present in the correct density within the trochanter major. Then as bones enlarge, the remodeling of the bones to retain proper orientation must occur. Altered trabecular bone volume, bone elongation, or reduced osteoclast function as suggested to accompany omega-3 supplementation [25, 29] would modify the joint formation. Altered joint conformation permissive for hip dysplasia and degenerative joint disease would reduce the quality of life for the dog. Therefore, this study assessed the impact of dietary DHA and EPA supplementation on joint conformation.

Overall, the proportion of dogs phenotypically considered dysplastic for hips was relatively low and substantially lower than that recorded for these breeds in the USA population (https://www.ofa.org/diseases/breed-statistics#detail accessed 6/1/2018). It is probable that the low proportion and absence of dogs with severe dysplasia reflects the concerted effort of the CCI to breed dogs with a low likelihood of dysplasia. This view is supported by the hip dysplasia heritability estimates being similar to those for a much larger USA population [41] and that the selection pressure applied at CCI is greater than that applied by breeders in the general public.

When assessing the potential influences on hip conformation, there were no effects of litter size, parity, or breed. The absence of an effect of litter size on hip conformation has been previously reported [42, 43] as has the lack of effect of dam age or number of previous litters [44]. Environmental impacts on hip formation are known [43] and although the care, feeding, and exercise regimens were prescribed to the volunteers who raised the puppies, strict adherence to those guidelines is unknown. Thus, whereas the environmental component may be a confounding factor in these results, the impact was managed by the provision of detailed day-to-day care directions. The data here demonstrated a direct, positive correlation in assessment of the dysplastic condition between the two hip evaluation schemes in the United States schemes as was shown previously [34]. One would not expect perfect correlation between the two schemes as the one scheme evaluates nine components in an integrative subjective assessment and the other measures femoral head distance within the hip joint.

The high DHA and EPA diet coincided with a significant, albeit small, increase in the DI for males. What a nominal increase in DI means biologically is unclear, as the phenotypic assessment of anatomical changes associated with hip dysphasia was not affected. Previous studies [45] have shown that arthritic changes accompany increasing age regardless of the initial DI measured. That being the case, for working dogs such as the population under study, early detection of physical changes associated with dysplasia is most germane to their physical longevity in their respective tasks.

The diets in the present study were formulated targeting DHA and EPA and while the diets were balanced for most other ingredients, there were differences in the level of vitamin D and E. The vitamin D levels across the three diets ranged from 1620 to 2330 IU/kg all within the upper limit established by NRC, AAFCO (Association of American Feed Control Officials), and FEDIAF (European Pet Food Industry Federation) [46]. Similarly, although vitamin E modestly differed across the diets (365–440 IU/kg), the levels were still well within the range defined by AAFCO. Supplementation of extremely high levels of vitamin D at 100 times the recommended level alters bone remodeling and enlarges growth plates in growing puppies [47] whereas supplementing puppies from 6–21 weeks of age with vitamin D at 100 µg/kg corresponded to normal bone growth consistent with that observed with a vitamin D supplementation of 12.5 µg/kg [48]. The vitamin D supplementation provided in the current study was therefore not expected to have influenced skeletal growth or joint formation. Vitamin E has been shown to increase bone formation in growing chicks when supplemented at three times the requirement [26, 27] well beyond the variance in diets of the present study. Thus, although the highest levels of both vitamins in the high diets had the potential to confound the interpretation of the omega-3 effects on joint formation, it is unlikely given past study results.

The finding that the OFA hip assessments were not statistically different between the diets would therefore suggest that the dietary regimen known to improve cognition and learning in dogs [15, 16] will not negatively impact structural soundness. A caveat to this assumption centers on the age of assessment. The hip joint scoring was done at 18 to 20 months of age preceding the recommended 24 months for OFA but at an acceptable age for PennHip. Although CCI does not formally monitor the orthopedic status of dogs throughout their lifetime career, the staff are alerted if dogs are retired from the program for dysplastic conditions; for the dogs in this study, there were no indications that this was the case. Furthermore, previous work for the breeds under study has demonstrated that DI values obtained at similar ages correspond to very low probability for degenerative joint disease at older ages [49] suggesting that the age of assessment did not impact the findings.

Interestingly, Smith et al. [45] suggested that DI evaluation of hip conformation is resistant to environmental influence and is a pure measure of genetic input. The data of the present study is not consistent with that view but rather indicates that dietary supplementation can influence measured DI indices confirming substantive environmental influences on hip conformation [50, 51]. The data suggest that omega-3 status may influence expression of genes involved in bone development. A recent study in fish demonstrated that elevated DHA increased larval growth and enhanced directly the expression of *IGF-I*, a gene highly involved in linear bone elongation [52]. Importantly, that study also demonstrated that excessive supplementation resulted in skeletal malformation; the authors posited that malformation was due to lipid peroxidation. The slight influence of the omega-3 on the DI is consistent with an effect on skeletal growth, a finding supported in the literature (reviewed in [53]) though the levels supplemented here did not result in adverse development as previously reported in adolescent dogs fed aldehydes as a proxy for oxidized lipids [25].

There was no sex effect on the OFA scores for the pups from different diets consistent with previous results [44, 49, 54], although other published studies using the nine-point radiographic scoring have shown females having higher scores associated with hip dysplasia [55, 56] sometimes in a breed specific manner. Transient increased joint laxity has been suggested to be associated with the estrous cycle [57, 58] though not with changes in DI [59]. The greater proportion of females receiving poorer scores in the literature is consistent with the females here having a significantly greater DI, independent of breed. The observation that the high DHA and EPA diet only influenced DI in the males may reflect that females are already expressing the maximal DI potential and additional growth effectors, such as dietary omega-3 fatty acids, were unable to further influence bone. Alternatively it may reflect that females had higher basal levels of circulating DHA than males, as is seen for humans [60]. The relationship of greater DI values for females has not been previously reported and suggests additional studies are warranted to uncover the underlying contributing factors of sex hormones on DI.

## Conclusion

The data presented here suggest that maternal dietary supplementation of DHA and EPA during pregnancy and the early perinatal growth period does not negatively influence joint formation of the offspring, further supporting the utility of supplementation for the neurological and cognitive benefits. As an ancillary finding, the data demonstrated a direct, positive correlation in assessment of hip dysplasia between the two USA evaluation schemes.

## Supporting information

S1 Table Raw data. (XLSX)
